# 

**Published:** 1956-07

**Authors:** 


### Contents

| | | Page |
|---|---|---|
| MEDICAL WORTHIES OF THE WEST COUNTRY | *Dr. P. Phillips* | 87 |
| THE GROWTH OF HUMAN TUMOURS IN LABORATORY ANIMALS | *Mr. G. J. Hadfield* | 94 |
| DOMESTIC DEATHS FROM ELECTROCUTION | *Dr. F. E. Camps* | 96 |
| SPACE MEDICINE | *Dr. S. G. Flavell Matts* | 103 |
| HYPERCALCAEMIA WITH HYPOCALCURIA, AN UNUSUAL CASE OF PRIMARY HYPERPARATHYROIDISM | *Dr. H. A. W. Forbes and M. J. Wodzinski* | 107 |
| GENERALIZED OSTEITIS FIBROSA WITH PARATHYROID ADENOMA | *Clinical Pathological Conference* | 112 |
| NOTES AND NEWS | | 115 |
| APPOINTMENTS | | 116 |

